# Mr. Macilwain on Stricture of the Rectum

**Published:** 1830-07-01

**Authors:** 


### XVIII.

On Stricture of the Rectum. By Mr. Macilwain. (2d Edition of his Work on Strictures.)

Mr. M. considers a protracted residence of fæcal matter in the rectum as always the result of disorder—and too frequently the precursor of disease. The muscular power of the gut is very considerable, and, in the ejection of its contents, receives the combined assistance of other muscles ordinarily engaged in respiration.

" The large calibre of this bowel, instead of being designed as some have imagined, to provide for a certain accumulation of fæces, seems to me to have been given with a very opposite intention, viz. as affording a facility to their descent through a situation, where, from the probable previous absorption of any nutritive fluids, their sojourn could no longer be useful."

The rectum is further endowed with a peculiar sensibility which pretty constantly keeps up a nisus to evacuate its contents, when they have increased to any considerable amount. Still, however, this sensibility may be greatly impaired by long habits of resisting the impulses of Nature, and then the presence of even large quantities of fæces in the rectum occasions little uneasiness. In this way the over-distended bowel loses more or less of its expulsive power, in the same way as the distended urinary bladder. Mr. M. is strongly impressed with the opinion that constipation, from negligence, is a common cause of stricture in the rectum.

" The causes of stricture in the rectum may be divided into those which result from disorder originating in the bowel, those which depend on its juxta position to other organs, and into those which are derived from more distant sources, by sympathy or otherwise. With regard to the causes which refer to the rectum individually, I believe the irregular performance of its functions to be the most frequent, whether this be induced by a torpid condition of the bowels generally, or by that gradual accumulation of fæcal matter, which takes place in the manner to which I have already adverted. Drastic purgatives frequently produce great irritation in the rectum, and they exert a still more noxious influence when that bowel is previously disordered by accumulated fæces or otherwise. The causes which depend on its sympathy with, or juxta position to, surrounding parts, refer to disease or irritation existing in the urinary or genital organs. Affections of the urethra, prostate, and bladder of the male, or of the vagina, uterus, and bladder in the female, are capable of inducing disease of the rectum. A retroverted or otherwise altered position of the uterus, and enlargement of the prostate gland, in consequence of their anatomical relation, sometimes give rise mechanically to obstruction in the rectum, without producing disease. As the different divisions of the alimentary canal sympathize with each other, so any disorder of the stomach or bowels may be productive of irritation in the rectum."

A loaded condition of the venous system generally, produces congestion of the hæmorrhoidal vessels particularly—thence arise piles, irritation, and disposition to stricture.

" Examination of strictured recta presents one or other of the following appearances: there is always more or less thickening and induration. Sometimes the thickening cannot properly be referred to any specific disease; frequently it is of a carcinomatous structure. It is either confined to a small space presenting an aperture, the edges of which are acute, which aperture may be of a rounded form or a mere slit, the bowel being free on either side of it, or the contraction occupies an inch or two, and there is a general thickening of the bowel apparently commencing in its muscular structure. To these appearances may be added ulceration of the stricture or bowel, or both. It is not uncommon to find the neighbouring surface of the bowel covered with firm flesh, like prominences such as might be supposed to result from hæmorrhoids, the blood of which had been absorbed. Piles and fistulæ not unfrequently exist in connexion with these appearances, of which there is a very fine specimen in the museum of the college. The situation of the contraction is generally within reach of a long finger, sometimes higher, even to the extent of several inches. Strictures have been found in the sigmoid flexure of the colon, but they are very rare, and, certainly, those who fancy that they have removed strictures in this part, must, I think, have deceived themselves. Stricture *in* the sigmoid flexure appears to me wholly out of the reach of surgery; but if the contraction be *at* the sigmoid flexure, that is, at the upper extremity of the rectum, it is certainly accessible by a bougie."

### TREATMENT.

This is divided into general and local. The general treatment comprehends affections of various viscera complicated with or causing the disordered rectum.

"The local treatment, like that of stricture of any other mucous canal, consists of the periodical introduction of instruments, preceded and accompanied by the employment of measures calculated to allay irritation. The former have for their object either dilatation, division, or destruction of the stricture, by which last I mean the application of caustic. The cases which have fallen under my care have been one or other of the following varieties. The obstruction has either been the result of malignant disease, an intus-suscepted condition of the rectum, a stricture consequent on common thickening, relievable by the common bougie, or so near the orifice as to admit of safe division. I have, therefore, never had occasion to employ either metallic instruments or bougies armed with caustic. No doubt there may be cases in which the latter mode of treatment might be employed with advantage, but I can only speak of those with which I have been practically acquainted. I shall, therefore, at once describe the measures which are calculated to relieve irritation, and the circumstances to which it is necessary to attend connected with the introduction of a bougie. As the irritation is to be relieved precisely in the same manner as when the stricture is in the urethra, it will be sufficient to enumerate the principal agents which in different cases we employ for this purpose. The catalogue consists of the local abstraction of blood by leeches at the verge of the anus, or cupping over the sacrum, blisters in the latter situation are sometimes of service; tepid emollient enemata, warm bathing, sitting over the steam of boiling water, occasionally a suppository, all likewise tend to diminish or relieve pain and irritation. It is desirable that the rectum should be kept as nearly as possible exempt from fæcal accumulations, and where the bowels have been previously cleared by mild purgatives, in conjunction with enemata of warm water or mucilage, the injections alone will be frequently found sufficient."

Mr. M. thinks that, in almost every case, the practitioner will find it advantageous to preface the introduction of the bougie by a mild aperient, assisted at a proper time by injections of warm water. In using the bougie, the same gentleness is necessary as in strictures of the urethra. In the majority of cases the contraction can be felt by the finger —that is, it is within a few inches of the orifice, so that a straight bougie will answer the purpose. But as, in all cases, it is desirable to ascertain the state of the whole of the rectum, our author prefers a curved instrument.

"With regard to the time which the bougie should be allowed to remain, the best rule is, to regulate it by the feelings of the patient, since, if it excite much pain it will do harm, whilst in the absence of any suffering, the longer it be allowed to remain (in reason) the better. I have seldom allowed more than seven or eight minutes at first, but have usually increased the length of time to a full hour, after two or three introductions. We shall seldom find it practicable to increase the size of the instrument with the same rapidity here, as in affections of the urethra; on the contrary, it will be generally necessary, and almost always expedient, to introduce the same instrument twice or thrice before we employ one of a larger diameter, allowing an interval of two or three days to intervene. Here, as in the corresponding affection of the œsophagus, the surgeon must bear constantly in mind, notwithstanding the absence of those circumstances which I have spoken of as indicative of malignant disease, that the malady may be of a carcinomatous nature, and be especially careful that he do not rouse a comparatively dormant disease into a frightful activity. If the introduction of the bougie is to be successful, the patient will soon experience considerable alleviation of suffering."

If the bougie is to be successful, the patient will soon experience considerable alleviation of his sufferings. The evacuations will either take place by the natural efforts of the bowels, or the bowels will be more amenable to medicinal influence. Occasional recourse should be had to the instrument after the full-sized one has been passed.

"Sometimes the stricture may be felt by the finger, and if its whole length can be judged of, and the accompanying symptoms are fairly explained by the degree of obstruction which it offers, its division may be safely performed, and the patient happily relieved in a much shorter time than by any other method. The mode of accomplishing this is by the introduction of a common probe-pointed bistoury, or that recommended by Sir Astley Cooper for strangulated hernia. I have always employed the former. It should be introduced lying flat on the finger, against which it should be firmly pressed; this will avoid the risk of its injuring any parts inferior to the stricture. When the finger has reached the contraction, the edge of the bistoury should be turned towards the side on which we propose to divide, the instrument being kept steady by the finger on which it has been introduced. As the hæmorrhoidal vessels frequently become enlarged under circumstances of continued irritation, it is better to divide the stricture by two or three very small incisions in different directions, than to accomplish its division in one. If the latter mode were preferred, a sacro-lateral direction would probably be the most safe; but as the practice above recommended will always effect the desired object, it should be preferred. As soon as the aperture be sufficiently enlarged to freely admit the finger, the case may then be treated by the bougie in the ordinary manner. I can confidently assert that this practice, adopted under favourable circumstances, has been productive of great and much accelerated relief, and that too in cases, in which, before the division of the stricture, bougies had made little or no impression. My friend, Mr. Kingdon, has also treated strictures of the rectum in this way with good success. Were it my object to speak of diseases of the rectum generally, I should have to shew, that as the irritation in the rectum which precedes stricture is attendant on piles, fistulæ, abscess, &c., so may these diseases be productive of symptoms simulative of those characterizing contraction of the bowel. This was not my intention. I cannot, however, quit the subject without mentioning the irritable sphincter, the symptoms of which so much resemble those of stricture. I believe it to depend on irritation in the bowel. The sphincter in this case resists the introduction of the finger with considerable power, and the irritation in the bowel frequently gives rise to painful efforts to eject the finger or bougie when brought in contact with it. The case may be known by the opposition afforded by the sphincter, and by the rectum being free from contraction. Attention to the general health, mild and spare diet, with regulation of the bowels, including the use of enemata, are in general successful."

We shall notice Mr. M's observations on stricture of the œsophagus in another article. We may here mention that the work from which we have analyzed the above, is a second edition of the author's former publication on urethral strictures, with the addition of a considerable portion of new matter.

